# “Heads Up” for Creatine Supplementation and its Potential Applications for Brain Health and Function

**DOI:** 10.1007/s40279-023-01870-9

**Published:** 2023-06-27

**Authors:** Darren G. Candow, Scott C. Forbes, Sergej M. Ostojic, Konstantinos Prokopidis, Matt S. Stock, Kylie K. Harmon, Paul Faulkner

**Affiliations:** 1 Aging Muscle & Bone Health Laboratory, Faculty of Kinesiology & Health Studies, University of Regina, 3737 Wascana Parkway, Regina, SK S4S 0A2 Canada; 2 Department of Physical Education Studies, Brandon University, Brandon, MB Canada; 3 Department of Nutrition and Public Health, University of Agder, Kristiansand, Norway; 4 Department of Musculoskeletal Biology, University of Liverpool, Liverpool, UK; 5 School of Kinesiology and Rehabilitation Sciences, University of Central Florida, Orlando, FL USA; 6 Department of Exercise Science, Syracuse University, New York, NY USA; 7 Department of Psychology, University of Roehampton, London, UK

## Abstract

There is emerging interest regarding the potential beneficial effects of creatine supplementation on indices of brain health and function. Creatine supplementation can increase brain creatine stores, which may help explain some of the positive effects on measures of cognition and memory, especially in aging adults or during times of metabolic stress (i.e., sleep deprivation). Furthermore, creatine has shown promise for improving health outcome measures associated with muscular dystrophy, traumatic brain injury (including concussions in children), depression, and anxiety. However, whether any sex- or age-related differences exist in regard to creatine and indices of brain health and function is relatively unknown. The purpose of this narrative review is to: (1) provide an up-to-date summary and discussion of the current body of research focusing on creatine and indices of brain health and function and (2) discuss possible sex- and age-related differences in response to creatine supplementation on brain bioenergetics, measures of brain health and function, and neurological diseases.

## Key Points

**Table:** 

Long-term high-dosage creatine supplementation increases brain creatine stores.
Creatine supplementation can improve cognition and memory, especially in older adults or during times of metabolic stress (i.e., sleep deprivation).
Creatine supplementation improves aspects of recovery from traumatic brain injury in children and has the potential to reduce symptoms of depression and anxiety.
There is some evidence that creatine supplementation improves outcome measures in those with muscular dystrophy but not other neurological diseases or conditions such as Parkinson’s disease or amyotrophic lateral sclerosis.

## Introduction

Creatine is a nitrogen-containing compound endogenously produced and synthesized in the human body from reactions involving the dispensable amino acids arginine and glycine and the indispensable amino acid methionine in a two-step process primarily in the liver and brain [1, 2]. Alternatively, creatine can be exogenously consumed through habitual dietary sources such as red-meat and seafood [2] or through the ingestion of commercially manufactured creatine. Almost the entirety of creatine supplementation is in the form of creatine monohydrate [3], which is the most effective and bioavailable form of creatine for increasing plasma creatine levels, tissue creatine content (i.e., brain, muscle), and performance outcome measures [3]. To date, there are well over 1000 peer-refereed papers published on creatine supplementation [4]. The vast majority of evidence-based research has focused on the efficacy of creatine monohydrate and exercise (primarily resistance-type training) for improving measures of muscle/lean mass, muscle performance (including strength, endurance, and power), and recovery from sport or exercise in young healthy male adults [4]. Research involving young healthy female individuals and aging adults is limited. Thus far, the combination of creatine and resistance-type training has been repeatedly shown to improve measures of muscle performance in young healthy female individuals [5] and post-menopausal female individuals [6] but a recent meta-analysis failed to find a greater effect from creatine (compared to placebo) on lean mass accretion in female individuals (independent of age) [7]. This lack of benefit from creatine supplementation may be related to female individuals having higher initial intramuscular creatine stores [8], which may subsequently blunt their responsiveness to creatine supplementation [9]; no decrease in measures of muscle protein catabolism from creatine supplementation over time [10, 11]; and the possible influence that fluctuations in estrogen have on creatine homeostasis [5]. Thus, a possible sex-interference effect may exist in response to creatine supplementation, at least for measures of lean mass in female individuals across the lifespan.

Over the past decade, accumulating research has shifted its focus from skeletal muscle and sport/exercise performance to the clinical and health aspects of creatine supplementation, with and without exercise. One major focus area of this paradigm shift is the efficacy of creatine supplementation on brain health and function, which we have previously reviewed [12]. However, additional research involving creatine supplementation has been published since this comprehensive review paper and we did not discuss possible sex- or age-related differences in response to creatine supplementation on indices of brain health and function. Therefore, the primary purposes of this narrative review are to: (1) provide an up-to-date summary and discussion of the current body of research focusing on creatine and indices of brain health and function and (2) discuss possible sex- and age-related differences in response to creatine supplementation on brain bioenergetics, measures of brain health and function, and neurological diseases.

## Creatine and Brain Bioenergetics

Creatine plays a critical role in the optimal functioning of the human brain. Acting as a temporal and spatial high-energy phosphate-storage buffer [13], creatine maintains intracellular levels of adenosine triphosphate (ATP) during energy-demanding cerebral activities, which account for about 20% of the body’s energy consumption [12]. The high rate of brain metabolism is remarkably constant despite widely varying mental and motoric activity [14], implying a permanent need for high-energy compounds such as creatine. This is exemplified by the relatively high (and consistent) levels of total creatine (including creatine and phosphocreatine) at 4–5 mM in the human brain, although lower when compared with levels in skeletal muscle (35–40 mM) [15]. Any hereditary or acquired impairments in brain creatine homeostasis could have severe consequences for normal brain development and function. For instance, cerebral creatine deficiency syndromes, rare inborn errors of creatine metabolism that interrupt the formation or transportation of creatine, are characterized by global developmental delays, intellectual disability, seizures, autism-like behaviors, and movement disorders [16]. In addition, low brain creatine accompanies several neurodegenerative disorders, with a magnitude of creatine shortfall often corresponding to a disorder’s severity [17]. To maintain a steady creatine supply, the brain relies on different sources of creatine, including dietary intake of creatine-containing foods and endogenous production from the liver and brain cells [12]. The individual contribution of each of these three creatine sources remains unknown, and is likely influenced by the magnitude of dietary exposure to creatine (and precursor molecules) and the functionality of creatine transport and synthesis machinery inside and outside the central nervous system (CNS). Dietary creatine and creatine synthesized in the liver are transported to the brain through the blood–brain barrier (BBB) via the creatine transporter (CT1), a sodium- and chloride-dependent multi-pass membrane protein [12]. The adult CNS appears to have a limited capacity for creatine uptake from circulation [18], with CT1 at the BBB playing a pivotal role in regulating the creatine level as a major pathway in the brain [19]. Alternatively, brain cells widely express two enzymes (l-arginine:glycine amidinotransferase and *g*uanidinoacetate *N*-methyltransferase) necessary for creatine synthesis, both during development and in adulthood. This suggests that brain creatine levels could be partially independent of external sources, including dietary creatine consumption [20]. However, when cerebral creatine is low or limited, creatine supplementation can positively affect brain creatine levels in a number of neurological conditions [21–23] but not in others (for a detailed review see [24]), highlighting a rather complex regulation and cellular trafficking of creatine in the stressed brain. In addition, different cell types within the CNS appear to have dissimilar capacity for creatine synthesis and transport (for a detailed review, see [25]).

Several factors might affect the potency of dietary creatine to increase brain creatine levels. It appears that a critical barrier could be the CT1 protein, which is not an abundant component of the BBB capillaries [26]. In addition, CT1 is downregulated by exogenous creatine intake, leading to possible resistance or an attenuated response to the compound after prolonged consumption [27]. For this reason, the upregulation of CT1 function has been identified as an innovative course of action to facilitate creatine uptake, with several exotic agents and routes cataloged so far, including glucocorticoid-regulated kinases, mammalian target of rapamycin, ammonia, and Klotho protein (for a detailed review, see [28]). Apart from CT1, extended creatine intake could diminish cerebral creatine synthesis through the end-product repression, with L-arginine:glycine amidinotransferase protein expression down-regulated by creatine [29]. To overcome the lack of CT1 expression at the BBB and down-regulation of endogenous brain creatine synthesis, higher dosages of creatine may be required. There is evidence that high-dose creatine supplementation (e.g., ≥ 20 g/day) can yield increases in creatine levels in the brain over periods of several weeks and perhaps overcome a reduction in endogenous creatine synthesis inside the CNS [30], but the replenishment of the brain creatine pool is still slow and limited [25]. The net effect of a long-term, higher dosage creatine intake on CT1 activity, brain creatine uptake, and/or endogenous brain creatine synthesis remains to be determined. Interestingly, brain creatine levels can be augmented by consuming guanidinoacetic acid, a creatine precursor that can be taken up by the brain via additional transporters besides CT1 and become readily available for methylation to creatine [31]. Similarly, cyclocreatine is another analog of creatine that can passively transit across membranes and presumably bypass CT1 to improve brain bioenergetics in an experimental model of CT1 deficiency [32]. With expedited creatine uptake being a pivotal target for many brain conditions, the above findings are highly relevant for identifying and validating promising alternatives to traditional creatine supplementation in further research. Nevertheless, brain cells likely have an upper limit for creatine accumulation, with possible metabolic effects of supra-physiological levels of brain creatine currently unknown, independent of sex or age.

## Creatine and Cognitive Function

Cognitive function encompasses an array of cognitive domains including attention, executive function, decision making, memory, reasoning, perception, language, creativity, and knowledge. Based primarily on animal research, creatine has been purported to play an important role in energetically demanding cognitive tasks involving learning and memory [12]. For instance, creatine ingestion in aged C57Bl/6 J mice exhibited improved object recognition memory, decreased latency to initiate exploration of a novel environment and a greater forward locomotor activity assessed by a modified Hole Board Test [33]. These neurobehavioral improvements were associated with small reductions in 8-hydroxy-2-deoxyguanosine levels and hippocampal lipofuscin. In addition, altered expression profiling was observed through upregulation of genes involved in neuronal growth, neuroprotection, and learning in the creatine-treated group (i.e., brain-derived neurotropic factor; non-synaptic diffusion neurotransmission, hepatocyte growth factor, transforming growth factor beta-2) [33].

There is some evidence to suggest that the cognitive benefits from creatine may be sex specific. In female mice with Alzheimer’s disease, creatine ingestion (3% wet weight) for up to 9 weeks decreased escape latency, which was associated with increased spatial learning; however, male mice did not experience the same benefits [34]. Mechanistically, in female individuals, creatine ingestion increased transcription factor- and plasticity-related proteins, including cAMP-response element binding protein, IκB [an inhibitory component of nuclear factor kappa-light-chain-enhancer of activated B cells], and postsynaptic density protein 95, while it also decreased amyloid β trimers. Further, chronic consumption of creatine enhanced spatial memory and hippocampal bioenergetics in male C57BL/6 mice coupled with increased protein levels of calcium-calmodulin-dependent protein kinase II and postsynaptic density protein 95, and decreased IκB [35]. Importantly, inhibition of nuclear factor kappa-light-chain-enhancer of activated B cells attenuates structural changes associated with hippocampus remodeling, such as dendritic branching and neural growth [34]. Additionally, downregulation of nuclear factor kappa-light-chain-enhancer of activated B cells phosphorylates cAMP-response element binding protein [36], which is involved in memory formation. In contrast, although cAMP-response element binding protein was increased in the first 30 min post-creatine ingestion in C57BL/6 mice, no differences were observed compared to controls at 180 min [37]. In young female Wistar rats injected with lipopolysaccharide to induce cognitive impairment, administration of creatine upregulated mammalian target of rapamycin signaling, synapsin, and postsynaptic density protein 95 in the dentate gyrus, ameliorating partial learning and memory deficits [38]; however, in male Wistar rats, creatine supplementation failed to improve learning, memory retrieval, and neuronal apoptosis [39]. Moreover, sleep deprivation is well established to alter brain bioenergetics and negatively affect cognitive function [40, 41]. In male Sprague–Dawley rats, a diet supplemented with 2% creatine for 4 weeks reduced sleep need and homeostatic pressure [42]. Micro-dialysis revealed that a sleep deprivation-induced increase in extracellular adenosine was attenuated following creatine supplementation [42]. Collectively, results across studies involving rodents clearly show sex differences exist in response to creatine supplementations on cognitive function.

Beyond animal studies, there is a growing number of human clinical studies that have investigated the efficacy of creatine monohydrate supplementation on indices of cognitive function (reviewed in [12, 43, 44]). Results of individual studies are mixed with some studies demonstrating improvements in cognitive function [45], while others failed to show any benefit [46, 47]. In healthy elderly participants (16 male; 16 female; aged 68–85 years), improvements in memory (forward number recall, backward and forward spatial recall, and long-term memory) were found following creatine supplementation (20 g/day for 7 days) [45]. In contrast, supplementation with creatine (0.03 g/kg/day; ~ 2.2 g/day) for 6 weeks in young adults (6 men and 5 women; aged 21.0 ± 2.1 years) did not improve components of cognitive processing (code substitution, logical reason, math processing, running memory, memory recall) [46]. The contrasting findings reported in these clinical studies may be associated with differences in age and the daily dose of creatine which substantially differed between studies (20 vs 2.2 g/day). It is worth highlighting that basal and post-intervention brain creatine content were not measured to decipher any differences that could have contributed to the contradicting results. Furthermore, similar outcomes were found after supplementing creatine (0.3 g/kg/day; split into four doses) for 1 week [47], which failed to promote any benefits on executive function and verbal learning in young adolescents (10–12 years; 38 boys and 29 girls). Importantly, brain creatine content in the left dorsolateral prefrontal cortex, left hippocampus, and occipital lobe remained unchanged after creatine supplementation, suggesting that the dosage and/or duration of creatine was not adequate to elevate brain creatine levels. Overall, these inconsistent findings across studies may be associated with differences in methodology, including age, assessment method, and responsiveness to creatine supplementation. A limitation across studies was that sex differences in response to creatine were not assessed and this warrants future research.

It is well established that stress (including hypoxia, sleep deprivation, and mental fatigue) alters brain energetics and appears to influence the efficacy of creatine supplementation on cognition. During acute oxygen deprivation (10% oxygen for 90 min), creatine supplementation (20 g/day for 7 days) in young healthy individuals (10 men and 5 women; mean age 31 years) attenuated omission errors during a continuous performance test compared with placebo [48]. Lower corticomotor excitability was linked to reduced cognitive performance in the placebo group; however, this association was alleviated following creatine supplementation [48]. In addition, following a 90-min mentally challenging task in young healthy adults (10 men and 4 women; aged 24 ± 3 years) using a crossover design, a high dose of creatine (20 g/day) did not counteract decrements in short sport-specific psychomotor and cognitive Flanker task performance, but enhanced executive function measured by a prolonged Stroop test [49]. These changes were not explained by alterations in physiological and perceptual factors. Further, creatine monohydrate (8 g/day) for 5 days in young healthy adults (19 men; 5 women; aged 24 ± 9 years) reduced mental fatigue, following a Uchida-Kraepelin test [50]. Specifically, participants were instructed to perform a series of calculations of random numbers for 15 min, take a 5-min rest, then perform another 15-min task. Significant improvements in mental fatigue reduction were observed following creatine supplementation but not post-placebo. These improvements corresponded with lower and higher oxygenation and deoxygenation, respectively, of cerebral hemoglobin at the second examination in participants ingesting creatine. No significant interactions between placebo and time were found for both oxygenated and deoxygenated cerebral hemoglobin. These findings suggest that creatine monohydrate may mediate hemoglobin oxygenation in the brain, accounting for potential improvement in cognitive function during long calculation tasks.

Additionally, in male elite rugby players (*n* = 10) performing a sport specific skill-based test (passing accuracy) either after normal sleep (7–9 h) or in a sleep-deprived state (3–5 h), supplementing with a placebo or creatine at two doses (50 mg/kg and 100 mg/kg) 1.5 h prior to testing, impaired passing accuracy as a result of sleep deprivation was attenuated in the creatine conditions. Specifically, the repeated rugby passing skill was performed indoors and consisted of a 20-m sprint where at the 10-m mark players had to pass a rugby ball through a hanging hoop [51]. Moreover, young adults (*n* = 20; 17 male, 3 female, aged 21 ± 2 years) who supplemented with creatine (20 g split into 4 × 5 g doses) [52] experienced significant improvements in random movement generation, verbal and spatial recall, choice reaction time, static balance, and mood after 24 h of sleep deprivation compared with placebo.

In a follow-up study from the same researchers, healthy young male individuals (*n* = 20; age: 21 ± 2 years) were randomized to supplement with creatine (20 g/day) or placebo for 7 days [53]. Participants then completed tests of executive function, short-term memory, choice reaction time, balance, and assessments of mood at baseline and after 18, 24, and 36 h of sleep deprivation. Moderate intermittent exercise, which included stair climbing and step ups (2 × 5 min at 65% maximum heart rate with 3 min recovery), and walking (15 min), was performed each hour to ensure similar energy expenditure between groups. Although no changes were depicted at 18 and 24 h, after 36 h of sleep deprivation, the creatine group was superior to placebo on a random number generation task, while changes in tasks engaging with mood and effort remained unaffected. Last, salivary cortisol levels were not different between groups throughout the 36-h sleep deprivation period. These findings are in contrast to those reported by McMorris et al. [52], who found significant improvements in cognitive performance after 24 h of sleep deprivation. However, it is worth stating that the aforementioned differences could be ascribed to the distinct nature of the tasks. In both studies, no significant effects were displayed following creatine supplementation on a number recall test, though on the contrary, choice reaction time was improved previously [52]. These findings highlight that creatine monohydrate has a minor effect on cognitive performance under sleep deprivation that may be dependent on longer duration, the complexity of a task performed, and specificity of potentially affected brain regions due to sleep loss-induced stress.

Overall, there is some evidence that creatine supplementation can augment measures of cognitive function when brain bioenergetics are challenged, such as with sleep deprivation, mental fatigue, and hypoxia. However, these effects may be dependent on the cognitive function test performed and the intensity and duration of stress conditions. Furthermore, the effects of age and sex on the responsiveness to creatine supplementation during these stressful situations (sleep deprivation, mental fatigue, hypoxia) are unknown.

## Creatine and Traumatic Brain Injury

There is a high prevalence of traumatic brain injuries (TBIs), including concussion, with a significant number of individuals that have persisting symptoms and delayed neurodegenerative ailments. One purported therapeutic intervention in the treatment of TBIs is creatine supplementation [12]. Creatine may alter brain pathophysiology and neurometabolic events induced by TBIs [12, 54]. A primary hallmark of TBIs includes an uncoupling of energy supply and demand due to altered cerebral energy availability and injury induced cerebral blood flow [55]. Further, TBIs reduces brain creatine content [12, 44]. An increase in brain creatine content through exogenous supplementation has been purported to be protective prior to and potentially enhances recovery following TBIs [44].

Animal models have provided promising evidence that creatine supplementation prior to inducing TBIs may be neuroprotective [56]. For example, mice injected with creatine (3 mg/g/day) for 3 or 5 days before a controlled cortical contusion had a 21% and 36% reduction in cortical damage, respectively, compared with placebo [56]. Sullivan et al. [56] reported that dietary creatine (1%) for 1 month following an experimental TBI ameliorated cortical damage by 36% in mice and 50% in rats. The neuroprotective effects of creatine against ischemic and oxidative insults appear to be due to the maintenance of mitochondrial membrane potentials and the ability of the phosphocreatine system to act as a spatial and temporal energy (ATP) buffer [56]. Creatine attenuates the alterations in ATP, thereby reducing free radical production and intra-mitochondrial calcium (Ca^2+^) [56]. Scheff and Dhillon [57] randomized adult male Sprague–Dawley rats to either an enriched diet consisting of 0.5% or 1% creatine or a standardized diet for 2 weeks before a moderate controlled cortical contusion. Seven days after the injury, the creatine-fed groups had significantly more cortical tissue sparing in the ipsilateral hemisphere compared with rats on the standard diet. The 1% creatine-fed group had a greater, but not statistically different, tissue-sparing effect compared with the 0.5% creatine-fed group [57]. Furthermore, concussion and a TBI alter the release of the excitatory amino acid glutamate that over activates the *N*-methyl-d-aspartate receptor, leading to an excitotoxic cascade including increasing cellular calcium (Ca^2+^), neuronal death, damage, and dysfunction [12, 58, 59]. In a rodent primary embryonal hippocampal and cortical cell culture model (> 99% neuronal, < 1% glial) that was challenged by glutamate or H_2_O_2_, the presence of creatine (5 mM) enhanced cellular energy and bioenergetics, reduced oxidative stress, and attenuated the Ca^2+^ response to N-methyl-D-aspartate receptor stimulation [60]. In addition, creatine (300 mg/kg) immediately following a TBI protected against oxidative stress but did not affect seizures compared to placebo [61].

An open-label randomized controlled trial (RCT) in children and adolescents (*n* = 39: 1–18 years of age) with a TBI revealed that creatine supplementation (0.4 g of creatine/kg/day) for 6 months had several positive effects. Specifically, creatine reduced the duration of post-traumatic amnesia, intubation time, and intensive care unit stay, in addition to improving disability, good recovery, self-care, communication, locomotion, sociability, personality and behavior, and neurophysical and cognitive function [62]. Further, creatine improved post-traumatic headaches, dizziness and fatigue [63], dysarthria, and lingual problems of understanding [62]. A potential limitation was that no sex-based analyses were completed, nor did they provide sex-based participant characteristics. Given that female individuals experience a higher rate of TBIs and greater adverse symptoms compared with male individuals, additional research is needed to determine possible sex-related differences in regard to brain creatine metabolism, with and without creatine supplementation [64].

Overall, based primarily on animal and young adult research, creatine supplementation has the potential to help manage symptoms associated with TBIs. However, it is currently unknown whether sex- or age-related differences exist in response to creatine supplementation in the treatment of TBI.s

## Creatine and Neurodegenerative Diseases

Neurodegenerative diseases are disabling conditions that progress slowly [65]. Neurodegenerative diseases occur when neurons in the central or peripheral nervous system lose function and eventually die [65]. Although some treatments may relieve some of the physical or mental symptoms associated with neurodegenerative diseases, slowing their progression is not currently possible. As such, identifying efficacious treatments to help patients manage their symptoms is a high public health priority. Given that the phosphocreatine system plays a critical role in numerous cellular and energetic pathways [12], creatine supplementation has been purported as an effective countermeasure that delays the progression of neurodegenerative diseases [66, 67].

### Alzheimer’s Disease

Alzheimer’s disease (AD) is the most common cause of neurodegenerative dementia, resulting in progressive loss of memory, difficulty with speech and cognition, disorientation, and eventual death [68]. There is sound theoretical rationale for investigating creatine as a therapeutic intervention in individuals with AD. Previous investigations have observed altered regulation of brain phosphocreatine [69, 70], a reduction in creatine kinase [71], reduced brain creatine levels in patients carrying an allele for AD development [72], as well as a neuroprotective effect of creatine against β-amyloid toxicity [73]. Additionally, AD results in altered brain glucose metabolism, reduced blood flow and oxygen utilization, and impaired mitochondrial respiration [74]. This energy failure is a precursor to the development of AD symptoms [75]. As the conversion of creatine to phosphocreatine results in readily available energy, ergogenic drugs such as creatine are attractive therapeutic candidates for AD. However, to the best of our knowledge, creatine supplementation in AD has not been investigated in humans, and only minimally in animal models with mixed results. Snow et al. [34] fed AD mice a creatine supplemented diet for 8–9 weeks, finding that creatine resulted in spatial cognition improvements in female mice, but negative effects on spatial cognition in male mice. While the exact reason for the sex differences is unknown, the authors hypothesized that it may have more to do with inherent sex differences in the AD mouse model, rather than sex- or disease-specific effects of creatine. Similarly, creatine supplementation appeared to exert negative effects in a rat model of AD, with 10 weeks of creatine feeding resulting in a deterioration of learning and memory retrieval, with no protection against neuronal apoptosis [76]. As there is some evidence to suggest that β-amyloid alters brain creatine kinase to concentrations toxic to neurons, the authors hypothesized that the increased creatine supply caused creatine deposition in neurons, resulting in increased inflammation and apoptosis. However, more work is needed to further determine the effects of creatine in AD.

### Parkinson’s Disease

Parkinson disease (PD) is a progressive neurodegenerative disorder that is expected to affect ≥ 10 million people worldwide by 2023 [77]. In addition to motor impairments, such as resting tremors, slowness, rigidity, and impaired balance and gait [78], individuals with PD often experience apathy, mood swings, depression, and anxiety [79]. Oxidative damage and mitochondrial dysfunction are hallmark features of PD, and the phosphocreatine system is known to play an important role in these cellular processes [80]. Given the promising scientific rationale for creatine to enhance outcomes measures in adults with PD, there has been accumulating work in this area. Early research by Matthews et al. [80] showed that creatine supplementation produced significant protection against 1-methyl-4-phenyl-1,2,3,6-tetrahydropyridine-induced dopamine depletions in mice, leading them and others [81] to speculate that the use of creatine supplementation would slow the neurodegenerative process in PD. Despite this potential, subsequent RCTs conducted in humans with PD that compared creatine supplementation (alone or in combination with other compounds) to placebo have shown disappointing results [82–86]. Bender and colleagues [82] first concluded that creatine supplementation did not show an effect on dopaminergic function or on health-related quality of life (SF-36); however, depressive symptoms did improve.

In the early 2000s, the National Institute of Neurological Disorders and Stroke conceived the National Institute of Health Exploratory Trials in Parkinson Disease program to evaluate therapies to slow the progression of PD, one of which was creatine supplementation. The initial two publications from this initiative showed that creatine supplementation was well tolerated [84] and safe [85]; however, among patients with early and treated PD, creatine monohydrate for ≥ 5 years did not improve clinical outcome measures [86]. Finally, one study focused on PD patients with mild cognitive impairment that compared a combination of creatine plus coenzyme Q10 supplementation reported no improvements in the severity of the patients’ motor symptoms; however, cognitive function was maintained at 12 and 18 months in the creatine plus coenzyme Q10 group, whereas significant declines were observed in the placebo group [83]. Overall, there is little evidence to suggest that creatine slows the progression of motor system impairment in PD.

### Multiple Sclerosis

Multiple sclerosis (MS) is an immune-mediated disorder caused by damage to myelin-producing cells, resulting in compromised nerve transmission. Multiple sclerosis is about twice as prevalent in female individuals than male individuals, and this trend has increased over time [87]. While the initial symptoms of MS can be noted at any age, the usual onset of MS is between 20 and 40 years of age [88]. The symptoms associated with MS vary, but frequently include skeletal muscle weakness, fatigue, poor vision, and difficulty with balance [88]. Unfortunately, there is no cure for MS. Most adults with MS seek to manage symptoms through medications and physical and occupational therapy. A growing body of literature highlights the importance of exercise for patients with MS [89]; both aerobic and resistance training have been shown to enhance physical performance and perceived fatigue [90].

Patients with MS show lowered cardiac phosphocreatine concentrations [91], impaired brain creatine metabolism [92], and elevated creatine kinase in cerebrospinal fluid [93]. Given these alterations in creatine metabolism [67], it would seem logical that creatine monohydrate supplementation would help patients with MS. Surprisingly, only two studies have examined this topic. In the first study, Lambert et al. [94] conducted a randomized, double-blind, placebo-controlled trial to investigate the effects of creatine monohydrate (20 g/day for 5 days) on muscle metabolite concentrations and knee extension and flexion performance. Vastus lateralis muscle biopsies were taken to measure intramuscular phosphocreatine, free creatine and total creatine. Creatine supplementation had no influence on the changes in muscle creatine stores over time compared to placebo (phosphocreatine: pre-creatine: 78.0 ± 16.4 mmol/kg dry muscle, post creatine: 83.8 ± 14.7 mmol/kg dry muscle; pre placebo: 67.5 ± 18.6 mmol/kg dry muscle, post placebo 67.7 ± 17.5 mmol/kg dry muscle, *p* = 0.67; free creatine: pre creatine: 38.1 ± 2.0 mmol/kg dry muscle, post creatine: 39.8 ± 2.5 mmol/kg dry muscle; pre-placebo: 29.1 ± 3.7 mmol/kg dry muscle, post-placebo 39.2 ± 2.2 mmol/kg dry muscle, *p* = 0.06; total creatine: pre-creatine: 116.1 ± 16.5 mmol/kg dry muscle, post-creatine: 123.6 ± 14.4 mmol/kg dry muscle; pre placebo: 96.6 ± 19.6 mmol/kg dry muscle, post placebo 106.9 ± 18.0 mmol/kg dry muscle, *p* = 0.84). The authors noted that these non-significant changes in muscle creatine levels, which contrast with significant increases observed from creatine supplementation in healthy individuals, likely explain the lack of increase in lower-limb exercise capacity.

Mechanistically, there is some evidence that individuals with muscular dystrophy and mitochondrial myopathy have reduced skeletal muscle creatine transporter concentrations compared with healthy individuals [95]. While the effects of multiple sclerosis on creatine transport kinetics are unclear, it could be possible that reduced skeletal muscle creatine transporter concentrations attenuated the increase in intramuscular creatine from creatine supplementation [95]. The lack of muscle performance findings by Lambert et al. [94]) was later supported by the work of Malin and colleagues [95] who, utilizing a 14-day, double-blind, crossover trial with a 3-week washout period, reported that creatine supplementation did not enhance knee joint power. While these two studies do not provide support for patients with MS to supplement with creatine, it is important to recognize that the overall body of literature on this topic is minuscule. Given the high prevalence of MS and the physical and cognitive challenges it presents [88], additional studies in this area are sorely needed, particularly within the context of sex- and age-related differences in regard to brain bioenergetics and neurophysiology. In addition to muscle function measures, physiology and biochemistry studies are needed to determine if creatine supplementation influences the etiology of MS.

### Amyotrophic Lateral Sclerosis

Amyotrophic lateral sclerosis (ALS) is a neurodegenerative disease that is fatal. Amyotrophic lateral sclerosis is the most prevalent type of motor neuron disease and affects the motor neurons responsible for voluntary muscle control [96], resulting in a variety of symptoms that can be debilitating, such as muscle loss, progressive weakness, and difficulty speaking. The majority of medications for ALS are meant to lessen the severity of symptoms, pain, and fatigue, as there is currently no known cure [97].

Based on the accumulating body of research showing that creatine supplementation increases muscle performance and muscle accretion primarily in healthy individuals [98], creatine supplementation has been explored as a possible therapy for patients with ALS [99, 100]. Creatine supplementation may protect against neuron loss in the motor cortex and substantia nigra [101], as well as decrease oxidative stress [102] and mitochondrial dysfunction [103], potentially resulting in improved quality of life for patients with ALS. Indeed, there have been promising results in several animal studies, with multiple reports indicating that creatine supplementation improves motor performance, protects against neuron loss, and extends survival rate in mice [104, 105]. In addition to delaying motor deficits and extending survival, Andreassen et al. [106] observed that creatine supplementation reduced cortical glutamate concentration, which is thought to play a role in neuron degeneration and death. If so, these findings may provide some evidence for the potential mechanistic role for how creatine contributes to increased longevity and improved motor performance. However, not all animal models of ALS show positive effects from creatine supplementation as Derave et al. [107] failed to find a beneficial effect from creatine supplementation on muscle functional capacity or function in mice. Overall, despite the collective body of research showing positive effects from creatine supplementation in mice with ALS, results from RCTs in humans with ALS are not as encouraging. In patients (*n* = 28) with probable or definite ALS, Mazzini et al. [108] showed that creatine supplementation (20 g/day for 7 days) significantly increased knee extensor and elbow flexor strength and reduced fatigue in these muscle groups. However, the creatine dosing protocol resulted in reports of diarrhea and gastrointestinal distress which caused six participants to withdraw.

In contrast, a series of studies found no beneficial effect from creatine 5–10 g/day supplementation (independent of exercise) on measures of muscle strength of performance compared to placebo in individuals with ALS [109–111]. Similarly, when examining the effect of creatine supplementation in patients with ALS compared to a historical control group, Drory and Gross [112] reported no beneficial effects of creatine 5-g/day supplementation on respiratory function. However, the sample size was small (*n* = 5) as the majority of patients succumbed to their disease or were moved to a ventilator throughout the study duration. Further, it should be noted that patients in these studies were in an advanced stage of ALS. It is yet to be determined if creatine may result in improved outcomes if supplemented earlier in the disease process.

### Muscular Dystrophies

Muscular dystrophies are neuromuscular diseases that result in significantly reduced skeletal muscle free creatine and phosphocreatine stores [113, 114] and have been linked to lower creatine transporter protein content and impaired creatine uptake and release [115]. Given the central role of creatine in these pathologies, there have been numerous attempts to investigate potential therapeutic benefits of creatine supplementation in patients with both Duchenne and Becker’s muscular dystrophies. These diseases are X-chromosome linked, affecting primarily male individuals. Both are the result of mutations in the dystrophin gene and often result in progressive muscle weakness, difficulty walking, and eventual cardiac or respiratory failure [116]. A diagnosis of muscular dystrophy often results in a significantly reduced life expectancy, with fatal outcomes as early as the third decade.

Positive effects of creatine supplementation have been observed in mice. Passaquin et al. [117] observed that creatine feeding significantly reduced muscle necrosis after birth in a mouse model of Duchenne muscular dystrophy. This was particularly evident in fast-twitch muscle fibers, with no preservatory effect demonstrated in slow-twitch fibers. Additionally, creatine supplementation restored mitochondrial respiration capacity, which is often impaired in muscular dystrophy. Similarly, Louis et al. [118] also observed beneficial effects in the mouse model of Duchenne muscular dystrophy, with creatine supplementation resulting in a reduction of dysfunctional hypertrophy of fast-twitch fibers often observed in muscular dystrophies, potentially improving overall function despite no observable mitigation of disease progression. Creatine supplementation has also been demonstrated to be beneficial in the mouse model of fascioscapulohumeral muscular dystrophy, with observed increases in muscle mass, grip strength, and mitochondrial content [119]. However, these improvements were only evident when combined with an exercise intervention, suggesting that the combination of exercise and creatine supplementation may help to attenuate muscle atrophy and dysfunction in patients with fascioscapulohomeral muscular dystrophy.

As muscle weakness is a hallmark characteristic of muscular dystrophies, changes in muscle strength have been implemented as the main dependent variable in studies investigating the effect of creatine in patients with dystrophinopathies. Notable improvements due to creatine supplementation have been observed in boys with both Duchenne and Becker’s muscular dystrophies. Utilizing a randomized, double-blind, cross-over design, Tarnopolsky and colleagues [120] observed significant improvements in grip strength following 4 months of creatine supplementation in boys with Duchenne muscular dystrophy. Additionally, significant improvements in fat-free mass were observed following creatine supplementation. In a randomized, double-blind, cross-over design trial examining the effect of creatine supplementation on boys with both Duchenne muscular dystrophy (*n* = 12) and Becker’s muscular dystrophy (*n* = 3), Louis et al. [118] observed a significant improvement in strength and almost two-fold increase in time to exhaustion. Additionally, no change in joint stiffness was observed during creatine supplementation. This is notable, as joint stiffness increased by 25% during the placebo phase. Implementing a 6-month, double-blind, placebo-controlled design, Escolar et al. [121] observed strong trends towards improvement in strength and functional parameters in individuals with Duchenne muscular dystrophy. Although there was no statistically significant effect of creatine supplementation, the authors noted a disease-modifying effect, which likely did not reach statistical significance owing to the unanticipated strength preservation in the placebo group. In a 50-week, double-blind, cross-over RCT, Davidson et al. [122] observed that a daily nutritional supplement containing whey protein, β-hydroxy β-methylbutyric acid, glutamine, and creatine monohydrate 5 g resulted in observed improvements in a 6-min walk distance and decreased inactive minutes per day in boys with Duchenne muscular dystrophy. However, no meaningful improvements in body composition or quality of life were noted, and the results were ultimately deemed inconclusive because of the small sample size. Further, while promising, this study did not investigate the effects of creatine supplementation alone, thus it cannot be definitively determined whether creatine, β-hydroxy β-methylbutyric acid, or glutamine were responsible for the observed improvements.

Despite the positive impact of creatine supplementation on strength and physical function in patients with Duchenne and Becker’s muscular dystrophies, this does not appear to be the case in patients with type 1 myotonic dystrophy [123, 124] or type 2 myotonic dystrophy/proximal myopathy [125]. This contrast seems to indicate that the effects of creatine supplementation may be disease specific and positive results should not be inferred for other types of dystrophies. However, it is currently unknown if the benefits of creatine supplementation in these patient populations are age specific. Interestingly, beneficial effects of creatine supplementation have been observed in young boys with muscular dystrophies [114, 126, 127], with non-significant effects primarily reported in adults. Overall, the results from RCTs suggest that short- and moderate-term creatine supplementation is safe, well tolerated, and increases muscle strength in patients with muscular dystrophies [127].

### Charcot-Marie-Tooth Disease

Charcot-Marie-Tooth disease (CMT) is a group of inherited motor and sensory neuropathies that cause muscle atrophy and weakness in the hands and feet [128]. Charcot-Marie-Tooth disease is slowly progressive and incurable. While it is typically not fatal, most patients experience difficulty with muscle stiffness and gait because of foot drop and increased foot supination [129].

Three studies have assessed the effects of creatine supplementation in patients with CMT. Doherty et al. [129] used a double-blind, placebo-controlled, crossover design to examine the potential benefits of 1 month of creatine supplementation in patients with CMT disease type 1 (*N* = 34) and type 2 (*N* = 5). Their findings demonstrated no significant differences in activities of daily living scales, body mass, fat-free mass, or body fat percentage after creatine supplementation as compared with placebo. The same research group then published two studies aimed to test the hypothesis that creatine supplementation would enhance strength and myosin heavy chain content in patients with CMT when combined with resistance training [130, 131]. Utilizing a randomized double-blind design, Chetlin et al. [130] evaluated 20 patients with CMT disease who completed a 12-week, at-home, resistance training program. The authors reported that creatine supplementation did not enhance outcomes beyond the benefits observed with resistance training alone. Smith and colleagues [131] examined whether the combination of creatine supplementation and resistance training would increase the percentage of type I myosin heavy chain content composition, as well as whether myosin isoform changes would correlate with improved chair stand performance in patients with CMT. The results showed that, when combined with resistance training, creatine supplementation resulted in a decline in type 1 myosin heavy chain content and an increase in type II myosin heavy chain content. Moreover, these changes were associated with an increase in chair rise performance. While speculative, the data presented by Smith et al. [131] suggest that creatine supplementation may alter skeletal muscle protein synthesis, activate satellite cells, and myosin heavy chain isoform in patients with CMT. Given that there are a limited number of therapeutic treatment options available for patients with CMT, more work in this area is needed.

## Creatine and Mood Disorders

Mood disorders such as major depressive disorder (MDD), bipolar disorder, anxiety disorders, and post-traumatic stress disorder are leading contributors to disability worldwide, with recent estimates suggesting that roughly 5–6% of the world’s population experiences symptoms of such disorders at any one time [132]. Furthermore, research suggests that the prevalence of mood disorders increased by as much as 28% in 2020 owing to the effects of the coronavirus disease 2019 pandemic [132].

Importantly, current therapies for mood-related disorders often fail to adequately support patients. For example, clinical trials report that talking and behavioral therapies such as cognitive behavioral therapy significantly alleviate symptoms in only 43–50% of patients with MDD (see Wiles et al. [133]). Furthermore, a systematic review of 522 clinical trials reveals that antidepressant pharmacotherapies reduce symptoms in only 60% of patients, which compares quite poorly with the 20–40% response rate to placebo treatments [134]. These figures are due in part to the fact that ~ 28% of patients stop taking their antidepressants within the first month after prescription, while ~ 44% do so within the first 3 months, and ~ 73% do so within the first 6 months [135], largely because of the high prevalence of side effects including sexual dysfunction (71.8% of patients), weight gain (63.5%), and feeling emotionally numb (64.5%) [136]. Importantly, the most commonly prescribed antidepressants are those that act upon the brain’s serotonin system (selective serotonin reuptake inhibitors), and the efficacy of this class of pharmacotherapy has been brought into question by recent reports that state that mood disorders are unlikely to be associated with low levels of serotonin (e.g., Moncrieff et al. [137]). As such, there is a need to better understand the neurochemical mechanisms of mood disorders. While many studies have used neuroimaging techniques such as positron emission tomography and ^1^H-magnetic resonance spectroscopy (^1^H-MRS) to examine neurotransmitter systems such as serotonin [138], dopamine (e.g., Howes et al., [139]), glutamate [140], and GABA [141], the results of these studies have not significantly aided the development of novel therapies. However, many of these studies often ignore the importance of creatine.

This is partly because, when using the primary method for examining this organic compound in vivo (^1^H-MRS), researchers have often referenced their neurotransmitter of choice (quantified as neurometabolite concentrations) to creatine in order to ‘correct’ for concentrations of other transmitters/metabolites (e.g., Kumar et al. [142]). However, these ‘corrections’ were often performed on the basis that concentrations of creatine were considered stable across both brain regions and individual participants (e.g., Li et al. [143]), yet ^1^H-MRS studies have indicated that concentrations of creatine within the prefrontal cortex are influenced by factors such as drug use [144–146], and are associated with pathological conditions such as hepatic encephalopathy [147] and with neuropsychiatric conditions such as schizophrenia [148].

Importantly, when determining whether mood disorders are associated with concentrations of brain creatine, researchers are usually hindered by the ‘single-voxel’ nature of many ^1^H-MRS sequences. Specifically, prior to scanning, the researcher must carefully position a relatively large (i.e., 2–8 cm^3^) voxel onto a pre-defined region on the participant’s structural magnetic resonance image manually. This requirement not only introduces a slight confound in terms of minor variations in voxel placement between researchers and/or studies, it also creates spatial limitations that constrain the researcher’s ability to determine the relationship between symptomatology and neurometabolite concentrations in the entire brain. While newer ^1^H-MRS scan sequences have recently been developed to allow researchers to examine metabolite concentrations in multiple brain regions simultaneously, no such studies have yet utilized these sequences. Because of these issues with ^1^H-MRS, the relationship between concentrations of creatine in the brain and symptoms of mood disorders is not yet fully understood. For example, when using ^1^H-MRS, no difference was found between 19 individuals with a clinical diagnosis of MDD and 30 healthy controls in concentrations of creatine within the anterior cingulate cortex [149], or between 18 young individuals with MDD and 18 aged-matched healthy controls in thalamic creatine concentrations [150].

However, research indicates that concentrations of creatine within the frontal cortex may relate to symptoms of mood disorders. For example, Kondo et al. [22] report a significant negative relationship between concentrations of creatine within a voxel encompassing the bilateral frontal pole and ventromedial prefrontal cortex, and scores on the Children’s Depression Rating Scale in female individuals aged 13–20 years with MDD. Furthermore, Faulkner et al. [151] report a similarly negative relationship between concentrations of creatine within a voxel in the medial prefrontal cortex and depression scores on the Depression, Anxiety and Stress Scale in a sample of 84 male and female participants who had not received a diagnosis of MDD, but who exhibited a wide range of depression scores (which can be considered to be representative of the general population). Further, Yue et al. [152] report that a small sample of nine medication-free patients with social anxiety disorder exhibited lower concentrations of creatine in the left dorsolateral prefrontal cortex than nine healthy controls, indicating that concentrations of creatine within regions of the prefrontal cortex are likely also associated with symptoms of anxiety.

Based on these results, increasing levels of creatine in the prefrontal cortex (and perhaps in other brain regions) may help to alleviate some of the symptoms of depression and anxiety. For example, Dechent et al. [30] report that daily administration of creatine monohydrate 20 g for 4 consecutive weeks increased total brain creatine by 8.7% in a small sample of nine healthy individuals, while Lyoo et al. [153] revealed that daily supplementation of only creatine monohydrate 5 g for 8 weeks improved the antidepressant effects of the selective serotonin reuptake inhibitor escitalopram in 25 depressed female individuals. It is worth noting that Nemets and Levine [154] report that daily administration creatine monohydrate failed to augment the antidepressant effects of selective serotonin reuptake inhibitors, yet this is likely due in part to the fact that the authors compared the effects of (i) creatine monohydrate 5 g, (ii) creatine monohydrate 10 g and (iii) placebo in small groups of only five, four, and nine participants respectively, as well as the fact that these low doses of creatine were administered over only 4 weeks. Taken together, these results indicate that daily administration of at least 20 g of creatine monohydrate over 4 weeks, or a lower dose (5 g) for at least 8 weeks, is needed to alleviate the symptoms of MDD. Importantly, the BBB has a relatively low permeability for creatine into the human brain, owing in part to the complete absence of a creatine transporter on the feet of astrocytes that line the microcapillary endothelial cells [155]. Such low permeability may be a contributing factor as to why Kondo et al. [22] reported that daily administration of creatine monohydrate 10 g over an 8-week period resulted in only a 9.1% increase in creatine concentrations within the frontal lobes. Further work is therefore needed to determine the optimal dose and treatment course to produce antidepressant effects. In addition, understanding whether such effects rely upon increasing concentrations of creatine within only the prefrontal cortex, or in other brain regions or the whole brain, would help to optimize this treatment approach.

Being able to determine the mechanisms by which increasing creatine alleviates symptoms of mood disorders may also aid this endeavor. Because neuronal creatine is released from neurons following an action potential and is then taken back into the neuron via the creatine transporter, many researchers consider creatine to be a neurotransmitter [156]. As such, it may be that alterations in its functioning as a neurotransmitter can promote depression. Further, administration of creatine can increase levels of brain-derived neurotrophic factor, which is known to have antidepressant effects [157]. In addition, because ATP is used to convert creatine to phosphocreatine, low concentrations of creatine are associated with lower release of ATP from astrocytes, which is in turn considered to promote symptoms of depression and anxiety [158, 159].

No matter the mechanisms by which brain creatine is associated with mood disorders, it is important to consider the role of individual differences in this putative relationship, particularly because both age [160] and sex (e.g., Faulkner et al. [145]) are known to influence depressive symptomatology. For example, Lind et al. [161] examined healthy individuals aged 18–79 years, and revealed that creatine increases with age in a multitude of brain regions including the medial prefrontal cortex, dorsolateral prefrontal cortex, anterior cingulate cortex, hippocampus, and thalamus. Conversely, several research studies have reported no significant effect of sex upon concentrations of creatine within the medial prefrontal cortex, dorsolateral prefrontal cortex, anterior cingulate cortex, hippocampus and parahippocampus, insula, thalamus, inferior parietal cortex, primary and secondary motor cortices, temporal and occipital lobes, precuneus, and cerebellum [151, 162–164]. Interestingly though, Tayoshi et al. [165] did report that healthy male individuals exhibited slightly (yet significantly) higher concentrations of creatine than female individuals within their left basal ganglia. Because dopamine is a key neurotransmitter within this brain region, and because altered functioning of the dopamine system has been associated with depression and mood disorders [166], it may be important to examine whether individual differences in sex influence the relationship between creatine and symptoms of mood disorders.

In summary, research to date has indicated that low creatine function within certain brain regions, particularly the prefrontal cortex, may be associated with a greater likelihood of experiencing symptoms of depression and anxiety, and that increasing such function via administration of creatine monohydrate may alleviate these symptoms. However, clinicians’ ability to treat mood disorders would be improved by researchers obtaining a better understanding of the neural mechanisms by which altered creatine function can contribute to such disorders. Furthermore, because the BBB has such low permeability for creatine into the brain, it is vital that researchers and clinicians alike can determine the optimal dosage and course required to alleviate the symptoms of mood disorders via administration of creatine monohydrate. Finally, determining whether individual differences in age, sex, clinical diagnosis, and symptom severity can influence treatment response may help clinicians to alleviate the suffering associated with mood disorders.

## Conclusions

**Fig. 1:**
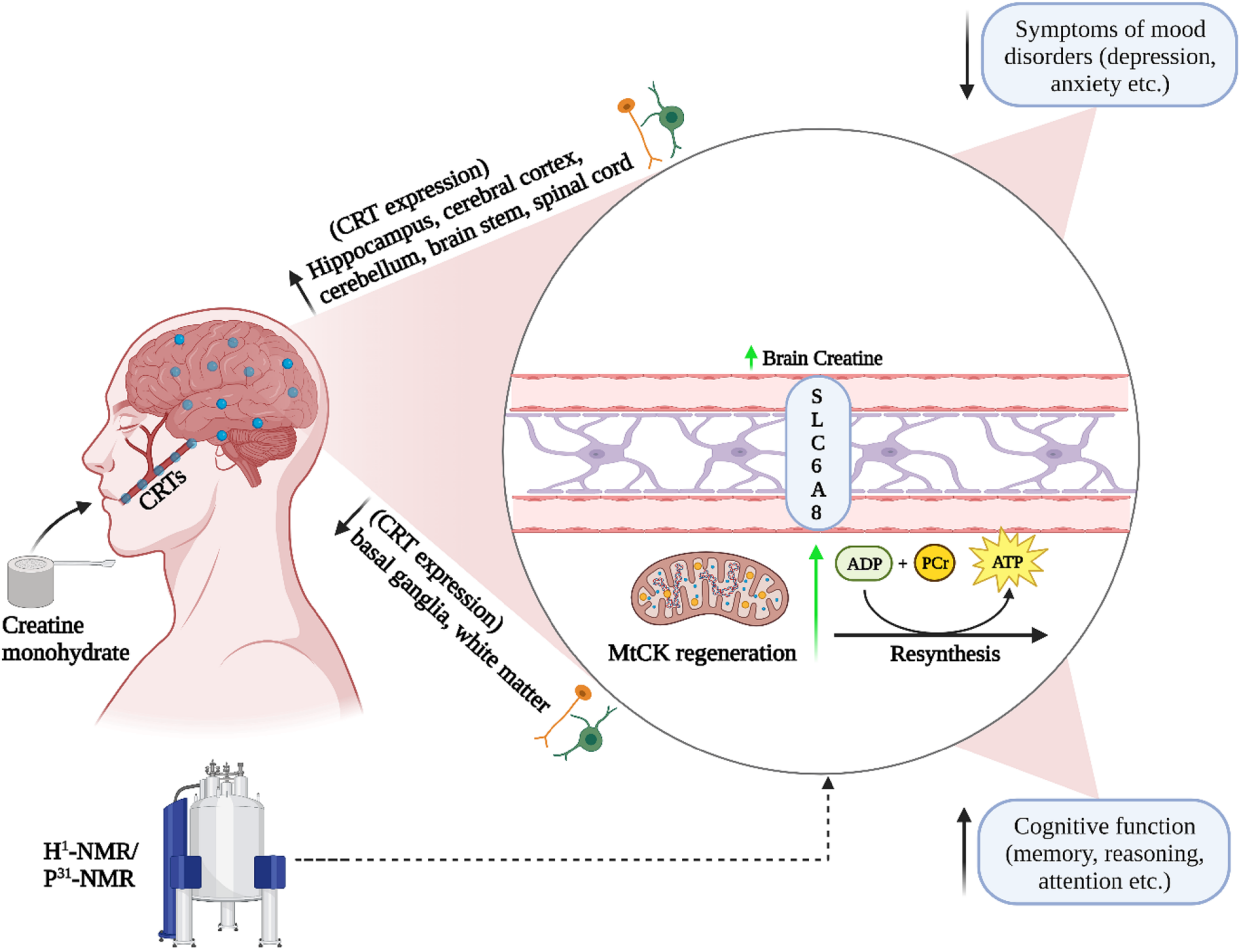
Potential effects of creatine monohydrate on measures of brain function. Creatine reaches the cytosol via CRT’s at the blood–brain barrier, neurons, and oligodendrocytes cells and contributes to the maintenance of glycolytic ATP levels. Creatine enters the mitochondria via MtCKs and converts ATP to PCr through oxidative phosphorylation. ATP and PCr are able to circulate from the mitochondria back into the cytosol, regulating energy requirements which in turn may enhance brain energy metabolism. ADP, adenosine diphosphate; ATP, adenosine triphosphate; CRT, creatine transporter; MtCK, mitochondrial creatine kinase; NMR, nuclear magnetic resonance; PCr, phosphocreatine

Creatine supplementation can increase brain creatine content, which over time may help explain some of the promising effects on measures of brain health and function (Fig. 1). Specifically, creatine supplementation has been shown to improve measures of cognition and memory (primarily in aging adults) and decreases symptoms of sleep deprivation in human and animal populations. Creatine supplementation also shows promise for alleviating some symptoms of TBI, including concussion, and characteristics of muscular dystrophy in humans. The efficacy of creatine for treating symptoms of depression and anxiety is also encouraging but clinical trials examining the effects of creatine (independent of pharmacological interventions) on these mood disorders are needed before a consensus can be reached. Future research is needed to determine the mechanistic effects of long-term creatine supplementation dosing strategies, with and without exercise, on brain function and health. Further, whether there are sex- and age-related differences in response to creatine supplementation remains to be fully determined.
