# Correction to: “Heads Up” for Creatine Supplementation and its Potential Applications for Brain Health and Function

**DOI:** 10.1007/s40279-023-01888-z

**Published:** 2023-07-10

**Authors:** Darren G. Candow, Scott C. Forbes, Sergej M. Ostojic, Konstantinos Prokopidis, Matt S. Stock, Kylie K. Harmon, Paul Faulkner

**Affiliations:** 1 Aging Muscle & Bone Health Laboratory, Faculty of Kinesiology & Health Studies, University of Regina, 3737 Wascana Parkway, Regina, SK S4S 0A2 Canada; 2 Department of Physical Education Studies, Brandon University, Brandon, MB Canada; 3 Department of Nutrition and Public Health, University of Agder, Kristiansand, Norway; 4 Department of Musculoskeletal Biology, University of Liverpool, Liverpool, UK; 5 School of Kinesiology and Rehabilitation Sciences, University of Central Florida, Orlando, FL USA; 6 Department of Exercise Science, Syracuse University, New York, NY USA; 7 Department of Psychology, University of Roehampton, London, UK

**Correction to: Sports Medicine** 10.1007/s40279-023-01870-9

Figure 1 was missing from this article. The figure should have appeared as shown below (Fig. 1)

**Fig. 1:**
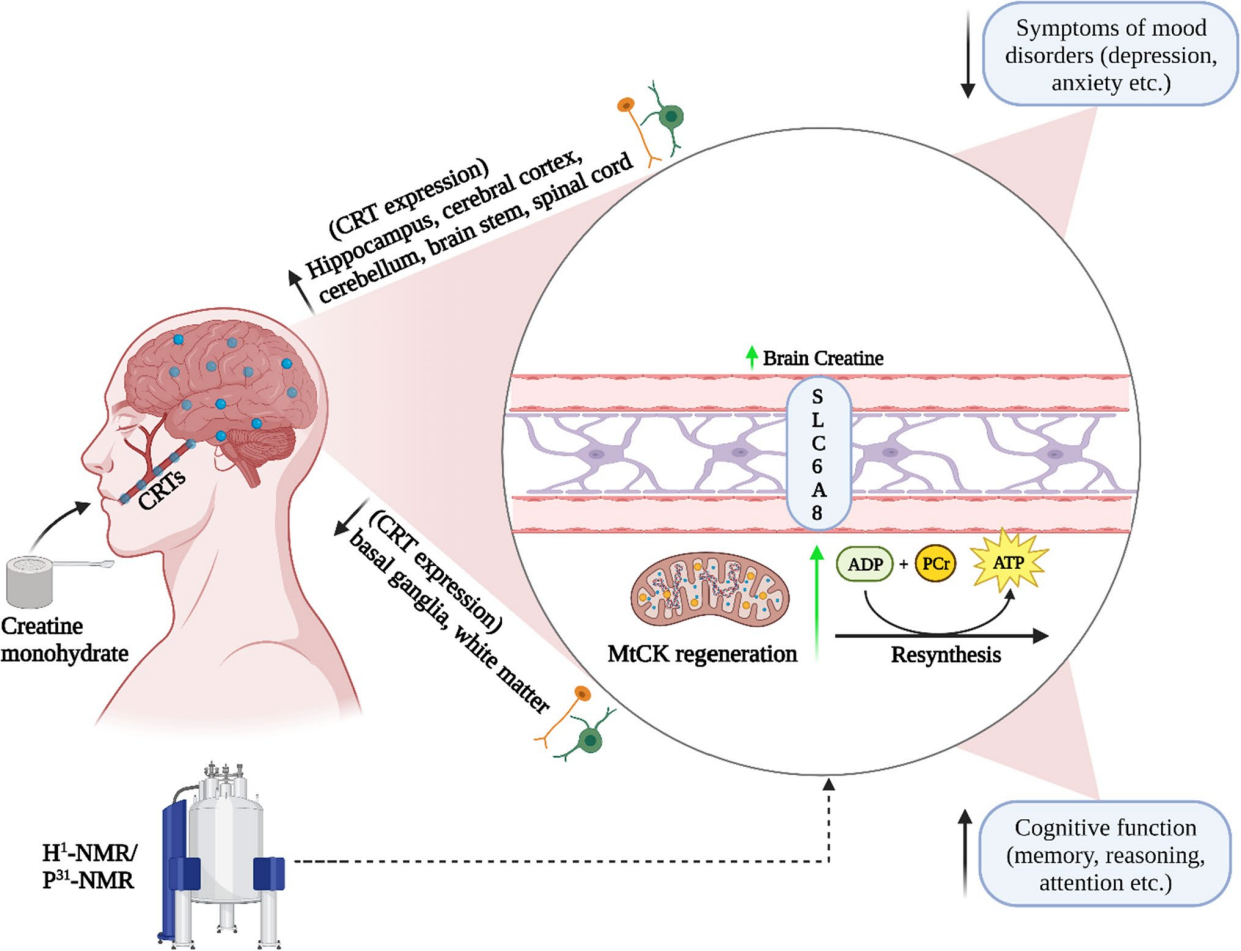
Potential effects of creatine monohydrate on measures of brain function. Creatine reaches the cytosol via CRT’s at the blood–brain barrier, neurons, and oligodendrocytes cells and contributes to the maintenance of glycolytic ATP levels. Creatine enters the mitochondria via MtCKs and converts ATP to PCr through oxidative phosphorylation. ATP and PCr are able to circulate from the mitochondria back into the cytosol, regulating energy requirements which in turn may enhance brain energy metabolism. (ADP, adenosine diphosphate; ATP, adenosine triphosphate; CRT, creatine transporter, MtCK, mitochondrial creatine kinase; NMR, nuclear magnetic resonance; PCr, phosphocreatine)

The original article has been corrected.

